# Targeting NAT10 with Remodelin in cancer drug resistance: mechanisms, preclinical evidence, and combination strategies

**DOI:** 10.3389/fphar.2026.1853392

**Published:** 2026-06-29

**Authors:** Feifeng Li, Wenzhi Deng, Xiulin Jiang, Zhi Li

**Affiliations:** 1 Department of Pathology, The Third Xiangya Hospital Central South University, Changsha, China; 2 College of Life Science, University of Chinese Academy of Sciences, Beijing, China

**Keywords:** ac4C RNA modification, cancer drug resistance, cancer therapy, DNA damage repair, drug resistance, epithelial–mesenchymal transition, Epitranscriptomics, immune checkpoint blockade

## Abstract

Drug resistance remains a major challenge in cancer therapy and limits the efficacy of chemotherapy, targeted therapy, and immunotherapy. Recent studies highlight RNA epitranscriptomic regulation, especially N4-acetylcytidine (ac4C), as an important mechanism in tumor adaptation. NAT10, the main enzyme that catalyzes ac4C formation, regulates RNA stability and translation. It plays key roles in cancer stemness, DNA repair, metabolic reprogramming, EMT, and immune regulation. Therefore, NAT10 has emerged as an important regulator of drug resistance in multiple cancer types. Mechanistically, NAT10 stabilizes key transcripts involved in DNA damage repair, metabolism, stemness, and EMT, thereby promoting tumor cell survival. It also enhances DNA repair by regulating DNA:RNA hybrid stability and homologous recombination, which reduces the effectiveness of DNA-damaging therapies. As a result, NAT10 contributes to resistance against cisplatin and other platinum drugs, doxorubicin, EGFR-TKIs, PARP inhibitors, and sorafenib. These resistance programs converge on a shared adaptive network involving DNA repair activation, metabolic rewiring, stemness maintenance, and immune escape. Remodelin is a small-molecule inhibitor of NAT10. It blocks ac4C modification and reverses drug resistance through multiple mechanisms. These include inhibition of DNA repair, suppression of metabolic adaptation, reversal of EMT, and improvement of the immune microenvironment. In preclinical models, Remodelin enhances the efficacy of chemotherapy, targeted therapy, and immunotherapy. However, clinical translation remains limited by issues such as off-target effects, toxicity, mechanistic complexity, and feedback regulation. Future work should focus on developing more selective NAT10 inhibitors, optimizing combination therapies, and identifying predictive biomarkers. Overall, NAT10 represents a potentially actionable regulator of cancer therapy resistance in selected tumor contexts, while Remodelin remains a useful preclinical tool for exploring NAT10-targeted strategies. Further validation is required before NAT10 inhibition can be considered a clinically applicable approach.

## 1 Introduction

Cancer drug resistance is one of the main reasons for treatment failure, tumor recurrence, and poor patient outcomes (Degirmenci et al., 2021). This problem is especially evident in the era of widely used chemotherapy, targeted therapy, and immunotherapy (Degirmenci et al., 2021). In recent years, it has become clear that drug resistance is not driven by single gene mutations alone. Instead, it is a complex adaptive process controlled by multiple molecular networks (Flaherty et al., 2019). These include enhanced DNA damage repair, metabolic reprogramming, cancer stem cell maintenance, epithelial–mesenchymal transition (EMT), and immune microenvironment remodeling. In this regulatory system, RNA epitranscriptomic modifications have emerged as an important layer of control in tumor plasticity (Flaherty et al., 2019). N4-acetylcytidine (ac4C) is a key RNA modification generated by N-acetyltransferase 10 (NAT10) (Wang Q. et al., 2025). NAT10 regulates mRNA stability and translation efficiency and is involved in tumor cell growth, survival, and stress adaptation (Wang Q. et al., 2025). Increasing evidence shows that NAT10 is highly expressed in many solid tumors and hematologic malignancies, and is closely associated with poor prognosis and therapy resistance (Wang Q. et al., 2025).

Mechanistically, NAT10 reshapes several major cancer-related pathways in an ac4C-dependent manner. These include DNA damage repair pathways (such as RAD51, AHNAK, and PARP1-related signaling), metabolic reprogramming (serine metabolism, glycolysis, and lipid metabolism), cancer stemness maintenance (such as NANOGP8 and PGAM1), EMT programs (such as HMGA1 and KRT8), and immune evasion (PD-L1 expression and tumor microenvironment remodeling) (Wang Q. et al., 2025; Li C. et al., 2025). Together, these changes may contribute to resistance to multiple therapies, including cisplatin, PARP inhibitors, doxorubicin, EGFR-TKIs, and sorafenib, although the strength of evidence varies across tumor types and treatment settings (Li C. et al., 2025). In this context, the small-molecule inhibitor Remodelin has been identified as an effective suppressor of ac4C modification. It reverses drug resistance in various cancer models. In preclinical models, Remodelin has been reported to disrupt NAT10-associated RNA regulatory programs and weaken adaptive processes such as DNA repair, metabolic flexibility, EMT progression, and immune suppression. These findings suggest that Remodelin may enhance sensitivity to selected therapies in specific experimental contexts, although its broad clinical relevance remains to be established (Xiong et al., 2026). Therefore, a summary of NAT10-mediated resistance mechanisms and the functional role of Remodelin is important for understanding RNA epigenetic regulation in tumor adaptation and for developing new combination therapeutic strategies.

Although NAT10 has been increasingly recognized as a key acetyltransferase involved in ac4C-mediated post-transcriptional regulation, drug resistance should not be interpreted as a NAT10-centered process alone (Gu et al., 2024). Therapeutic tolerance arises from multilayered and context-dependent mechanisms, including compensatory signaling pathways, metabolic rewiring, chromatin remodeling, RNA-binding protein networks, stress-response programs, and changes in the tumor microenvironment. In this regard, NAT10 may function as an important regulatory node rather than a single dominant driver. More importantly, the therapeutic implications of ac4C biology extend beyond direct NAT10 inhibition. NAT10-dependent ac4C marks can influence RNA stability, translation efficiency, and stress-adaptive gene expression programs through cooperation with RNA-binding proteins, transcript-specific regulatory elements, and downstream effector pathways (Shi et al., 2023). Therefore, targeting NAT10-mediated ac4C modification may involve multiple strategies, including inhibition of NAT10 enzymatic activity, disruption of NAT10-associated protein complexes, blockade of ac4C-dependent RNA regulatory circuits, or combination approaches that target parallel resistance pathways. Such a broader framework may help avoid an overly narrow focus on NAT10 itself and support the identification of alternative or complementary therapeutic targets within the ac4C-regulated resistance network. Ultimately, a balanced understanding of NAT10-mediated ac4C regulation should integrate NAT10 with other epigenetic, transcriptional, metabolic, and microenvironmental mechanisms that collectively shape drug tolerance and therapeutic vulnerability.

## 2 Mechanisms of NAT10 in tumor drug resistance

### 2.1 NAT10 promotes cancer stem cell self-renewal and drug resistance

Cancer stem cells (CSCs) are considered a major source of tumor relapse and acquired drug resistance. They have strong self-renewal capacity, enhanced DNA repair ability, and high expression of drug efflux transporters (Zhang et al., 2026). Therefore, factors that maintain CSC properties often contribute directly to chemotherapy resistance (Zhang et al., 2026). Recent studies show that NAT10, the key enzyme that catalyzes ac4C RNA modification, plays an important role in maintaining CSC traits. In ovarian cancer models, NAT10 is significantly upregulated and is accompanied by increased ac4C levels. Functional experiments show that NAT10 knockdown reduces sphere formation ability and decreases stemness-related protein expression (Zhang and Shen, 2025). It also suppresses glycolysis, including glucose uptake, lactate production, and extracellular acidification. Mechanistically, NAT10 increases the stability of PGAM1 mRNA through ac4C modification. PGAM1 is a key glycolytic enzyme (Zhang and Shen, 2025). This process supports metabolic reprogramming and helps maintain stem-like properties. Re-expression of PGAM1 can partially rescue the effects caused by NAT10 loss, confirming its functional role. These results suggest a NAT10–metabolism–stemness axis that promotes tumor progression. In cervical cancer, NAT10 is also highly expressed in drug-resistant tissues and correlates with elevated ac4C levels. NAT10 maintains CSC features and reduces chemotherapy sensitivity (Gao et al., 2024). In contrast, inhibition of NAT10 using Remodelin increases drug response. Mechanistically, NAT10 stabilizes NANOGP8 mRNA through ac4C modification, supporting CSC maintenance and drug resistance (Gao et al., 2024). Overall, NAT10 promotes CSC self-renewal and metabolic adaptation by stabilizing key metabolic enzymes (such as PGAM1) and stemness factors (such as NANOGP8). This links RNA epigenetic regulation, metabolism, and stemness, making NAT10 a potential target to reverse drug resistance.

### 2.2 NAT10 shapes the immunosuppressive tumor microenvironment and immune resistance

TME plays a key role in drug resistance, especially in immunotherapy. An immunosuppressive TME, including T cell exhaustion, Treg enrichment, and reduced immune infiltration, leads to immune escape and treatment failure (Ma et al., 2025). NAT10 regulates the TME through ac4C-dependent control of metabolism, immune checkpoint expression, and cytokine networks. In pancreatic cancer, NAT10 stabilizes LAMB3 or ETS2 mRNA and activates FAK/ERK signaling, leading to increased PD-L1 expression (Chen et al., 2025). This suppresses CD8^+^ T cell activity and promotes immune evasion. Blocking NAT10 can restore T cell function and improve response to PD-1/PD-L1 therapy (Chen et al., 2025). Metabolic reprogramming is another important mechanism. In cervical cancer and triple-negative breast cancer, NAT10 increases expression of glycolysis-related genes through transcription factors such as FOXP1 and JunB. This promotes lactate accumulation, which enhances Treg function and suppresses effector T cells (Chen et al., 2023). NAT10 also affects immune cell recruitment. In prostate cancer, it suppresses CD8^+^ T cell infiltration through the CCL25/CCR9 axis. In head and neck cancer, NAT10 stabilizes GLMP mRNA and activates MAPK/ERK signaling, affecting both angiogenesis and immune cell balance (Liu J. et al., 2024). In addition, NAT10 can suppress innate immune activation. It inhibits dsRNA accumulation and type I interferon signaling via the MYC/CDK2/DNMT1 pathway, leading to an immune “cold” tumor state. Inhibition of NAT10 activates antiviral-like immune responses and improves response to PD-1 blockade (Liu et al., 2025). In summary, NAT10 promotes immune escape by increasing PD-L1 expression, enhancing glycolysis and lactate production, reducing CD8^+^ T cell infiltration, and increasing Treg activity. It may function as an important regulatory link between tumor-cell metabolism and immune modulation in certain tumor contexts.

### 2.3 NAT10-mediated DNA damage repair and resistance to DNA-damaging therapies

DNA damage repair (DDR) is a major mechanism by which cancer cells evade the effects of chemotherapy, radiotherapy, and PARP inhibition (Ma et al., 2025). Recent studies suggest that NAT10-mediated ac4C RNA modification may enhance DDR by regulating RNA stability, translation, and chromatin-associated repair processes (Chen et al., 2025). In bladder cancer, cisplatin induces NAT10 expression and increases ac4C levels through NF-κB signaling. NAT10 subsequently stabilizes AHNAK mRNA, thereby promoting DNA repair and cisplatin resistance, whereas NAT10 inhibition with Remodelin restores cisplatin sensitivity (Xie R. et al., 2023). In UV damage models, NAT10 regulates the stability of DDB2 mRNA, a key component of nucleotide excision repair, although this effect may vary depending on the type of DNA damage and cellular context (Yang et al., 2023). NAT10 also participates directly in DNA double-strand break repair. It can be recruited to damage sites in a PARP1-dependent manner, where it modifies RNA within DNA–RNA hybrid structures, stabilizes these structures, and facilitates homologous recombination repair (Xu et al., 2025). In addition, NAT10 may regulate DDR through protein acetylation, such as acetylation of MORC2, which promotes G2/M checkpoint activation and allows tumor cells more time to repair DNA damage. In colorectal cancer, NAT10 enhances the translation of MYC and PPAN through ac4C modification, forming a NAT10–MYC–PPAN axis that further strengthens DNA repair capacity (Wang et al., 2026). Collectively, these findings indicate that NAT10 supports DNA repair through multiple regulatory layers, including RNA stabilization, translational control, RNA modification at damage sites, and protein acetylation. Targeting NAT10 may therefore weaken repair capacity and sensitize selected tumors to DNA-damaging therapies.

### 2.4 NAT10-mediated metabolic reprogramming in therapy adaptation

Metabolic reprogramming enables tumor cells to maintain growth and survival under nutrient limitation, therapeutic pressure, and microenvironmental stress (Liu et al., 2024b). NAT10 contributes to this process by regulating the stability and translation of metabolism-related transcripts through ac4C modification. In acute myeloid leukemia, NAT10 increases the expression of SLC1A4 and HOXA9/MENIN, promoting serine uptake, serine biosynthesis, leukemia cell growth, and stemness (Zhang et al., 2024). NAT10 inhibition exposes this serine-dependent vulnerability and suppresses tumor progression. In osteosarcoma, NAT10 stabilizes ATF4 mRNA and increases ASNS expression, thereby activating the ATF4–ASNS–asparagine axis and supporting tumor-cell survival under metabolic stress (Zou et al., 2024). In cervical cancer, NAT10 enhances FOXP1 translation and activates GLUT4 and KHK, leading to increased glycolysis and lactate production (Chen et al., 2023). Because lactate can also contribute to immunosuppression within the tumor microenvironment, NAT10-driven metabolic remodeling may indirectly influence immune evasion as well as tumor-cell fitness. Overall, NAT10 promotes amino acid metabolism and glycolytic adaptation, providing cancer cells with metabolic flexibility during therapeutic stress.

### 2.5 NAT10-mediated EMT, invasion, and metastatic drug tolerance

Epithelial–mesenchymal transition (EMT) promotes tumor invasion, metastatic dissemination, stem-like properties, and reduced sensitivity to anticancer therapy (Shibue and Weinberg, 2017). NAT10 has been implicated in EMT regulation mainly through ac4C-dependent stabilization of EMT-associated transcripts and activation of related signaling pathways. In prostate cancer, high NAT10 expression correlates with advanced tumor stage and metastasis. Mechanistically, NAT10 stabilizes HMGA1 mRNA to promote cell-cycle progression and also stabilizes KRT8 mRNA to facilitate EMT-associated phenotypes. In gastric cancer, NAT10 inhibition reduces proliferation and invasion, suppresses AKT signaling, increases the epithelial marker E-cadherin, and decreases mesenchymal markers such as N-cadherin and Vimentin (Li et al., 2024). In non-small cell lung cancer, NAT10 promotes tumor growth and metastasis, whereas Remodelin-mediated NAT10 inhibition reverses EMT features and reduces tumor progression. Together, these findings suggest that NAT10 may enhance metastatic plasticity by regulating HMGA1, KRT8, AKT signaling, and EMT marker expression (Li et al., 2024). However, the extent to which these EMT-related effects directly drive clinical drug resistance remains to be further validated in patient-derived models and clinical samples.

## 3 Preclinical evidence for remodelin-mediated NAT10 inhibition in therapy-resistant cancer models

**FIGURE 1:**
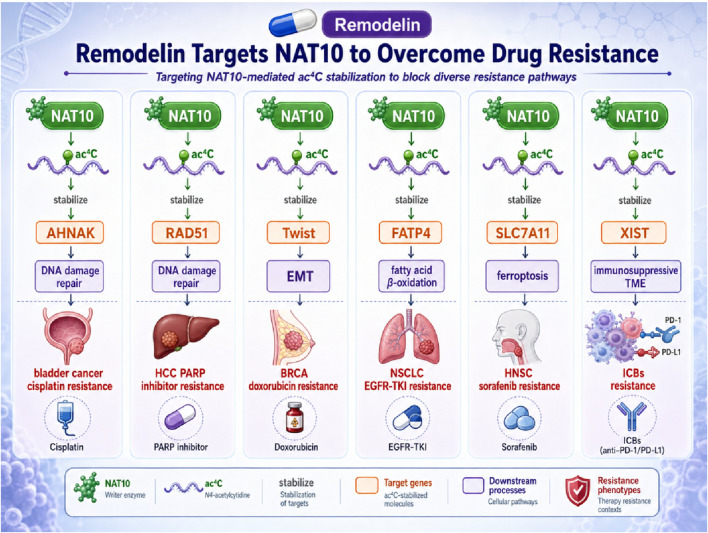
NAT10-mediated ac4C stabilization of target transcripts contributes to diverse therapy-resistance phenotypes.

Beyond these general considerations, accumulating preclinical evidence suggests that NAT10-mediated ac4C stabilization of specific target transcripts contributes to distinct therapy-resistance phenotypes across cancer types. These NAT10–ac4C-regulated axes involve DNA damage repair, epithelial–mesenchymal transition, fatty acid β-oxidation, ferroptosis regulation, and immunosuppressive remodeling of the tumor microenvironment. Representative examples linking NAT10-dependent transcript stabilization to resistance against chemotherapy, targeted therapy, and immune checkpoint blockade are illustrated in Figure 1.

### 3.1 Remodelin to overcome cisplatin resistance in cancers

Cisplatin is one of the most widely used platinum-based chemotherapeutic agents in clinical practice (Dasari and Tchounwou, 2014). It is commonly applied in bladder cancer, ovarian cancer, lung cancer, and head and neck cancers. Its main mechanism is the induction of DNA damage through DNA crosslinking, which triggers apoptosis in cancer cells (Dasari and Tchounwou, 2014). However, cisplatin resistance remains a major limitation that leads to treatment failure, tumor recurrence, and poor prognosis. The underlying mechanisms are closely associated with enhanced DNA damage repair, abnormal RNA epitranscriptomic regulation, and activation of pro-survival signaling pathways (Dasari and Tchounwou, 2014). Recent studies have shown that NAT10-mediated ac4C RNA modification plays a key role in cisplatin resistance. Its small-molecule inhibitor Remodelin provides an important strategy to reverse this resistance. In bladder cancer, cisplatin treatment induces increased ac4C levels and upregulates NAT10 expression (Xie R. et al., 2023). This process is closely associated with chemoresistance and poor clinical outcomes. Mechanistically, cisplatin activates the NF-κB signaling pathway. NF-κB p65 directly binds to the NAT10 promoter and enhances its transcription. This increases the adaptive capacity of cancer cells to cisplatin. Functionally, NAT10 stabilizes AHNAK mRNA through ac4C modification, significantly increasing transcript stability (Xie R. et al., 2023). AHNAK is a key regulator of the DNA DDR. It promotes DNA repair and improves the repair efficiency of cisplatin-induced DNA damage, leading to increased chemoresistance. Therefore, the NAT10–AHNAK–DDR axis represents an important mechanism of cisplatin resistance (Xie R. et al., 2023).

### 3.2 Remodelin to overcome PARP inhibitor resistance in cancers

PARP inhibitors (PARPi), such as olaparib, are important targeted therapies for tumors with BRCA mutations or homologous recombination deficiency (HRD) (Zeng et al., 2024). They are widely used in ovarian cancer, breast cancer (especially triple-negative breast cancer, TNBC), prostate cancer, and some liver cancers. However, clinical responses are often limited by acquired resistance (Zeng et al., 2024). Major mechanisms include restoration of homologous recombination repair, enhanced DNA damage repair, and increased replication fork stability. Therefore, strategies that enhance PARPi sensitivity are critical for overcoming resistance and improving patient survival (Zeng et al., 2024). Studies have shown that NAT10 plays a key role in DSB repair. In a PARP1-dependent manner, NAT10 rapidly accumulates at DSB sites. It catalyzes ac4C modification on DNA:RNA hybrid structures, increasing their stability and promoting homologous recombination (HR) repair efficiency (Xu et al., 2025). This enhances DNA repair capacity and reduces the cytotoxic effect of PARP inhibitors. In HCC, NAT10 loss or inhibition suppresses tumor progression and increases PARPi sensitivity. Mechanistically, NAT10 supports DSB repair by stabilizing DNA:RNA hybrids, thereby sustaining HR activation. This contributes to PARPi resistance (Xu et al., 2025). In TNBC, NAT10 stabilizes RAD51 mRNA through ac4C modification and increases RAD51 expression. RAD51 is a core HR protein. Its upregulation restores DNA repair capacity and leads to PARPi resistance (Li H. et al., 2025).

### 3.3 Remodelin to overcome doxorubicin resistance

Doxorubicin is a commonly used anthracycline chemotherapeutic agent. It is widely used in breast cancer, hepatocellular carcinoma, sarcoma, and other solid tumors (Saleem et al., 2025). It acts mainly by intercalating into DNA, inhibiting topoisomerase II, and inducing DNA damage (Saleem et al., 2025). However, its clinical efficacy is often reduced by acquired resistance, especially in breast and liver cancers. One important mechanism of resistance is EMT, which increases tumor invasiveness and chemoresistance (Saleem et al., 2025). Recent studies show that NAT10 is involved in doxorubicin resistance by regulating EMT, and its inhibition by Remodelin can reverse this process. In breast cancer, NAT10 is highly expressed in doxorubicin-resistant cells. Inhibition of NAT10 by siRNA or Remodelin significantly reduces cell viability and increases drug sensitivity (Wu et al., 2018). Doxorubicin induces EMT, characterized by decreased E-cadherin and increased Vimentin. NAT10 inhibition reverses this phenotype, restores epithelial characteristics, and reduces cell migration. Further studies show that Twist, a key EMT transcription factor, is essential for this process (Wu et al., 2018). When Twist is silenced, Remodelin loses its ability to reverse resistance, indicating that NAT10 regulates doxorubicin resistance through a Twist-dependent EMT pathway. In hepatocellular carcinoma, similar mechanisms are observed. NAT10 inhibition enhances doxorubicin sensitivity and reverses EMT induced by drugs or hypoxia (Zhang et al., 2019). Combination treatment significantly suppresses tumor growth and proliferation *in vivo*, showing strong synergistic effects. Overall, Remodelin reverses EMT and restores epithelial phenotype by inhibiting NAT10, thereby enhancing doxorubicin efficacy and providing a promising strategy to overcome anthracycline resistance.

### 3.4 Remodelin to overcome EGFR-TKI resistance

EGFR tyrosine kinase inhibitors (EGFR-TKIs), such as gefitinib, erlotinib, and osimertinib, are standard treatments for EGFR-mutant non-small cell lung cancer (NSCLC) (Zhao et al., 2024). They significantly prolong patient survival. However, almost all patients eventually develop acquired resistance, leading to disease progression and treatment failure (Zhao et al., 2024; Du et al., 2025). Resistance mechanisms include metabolic reprogramming, bypass signaling activation, and epigenetic alterations. Recent studies show that NAT10-mediated ac4C RNA modification regulates lipid metabolism genes and plays a key role in EGFR-TKI resistance. Remodelin may partially reverse this resistance in experimental models by inhibiting NAT10-associated regulatory programs. In NSCLC, NAT10 is highly expressed and associated with poor prognosis. NAT10 knockdown inhibits proliferation, induces apoptosis, and increases sensitivity to EGFR-TKIs both *in vitro* and *in vivo* (Fang et al., 2025). Mechanistically, NAT10 stabilizes mRNAs of lipid metabolism genes, including FATP4 and CPT1A, through ac4C modification. This enhances fatty acid uptake and β-oxidation, providing alternative energy sources under EGFR inhibition and promoting resistance (Fang et al., 2025). Upstream regulation studies show that p300-mediated H3K27 acetylation activates NAT10 transcription. Remodelin blocks the p300–NAT10–lipid metabolism axis, reduces metabolic adaptation, and restores EGFR-TKI sensitivity.

### 3.5 Remodelin to overcome platinum drug resistance

Platinum-based drugs, including cisplatin, carboplatin, and oxaliplatin, are widely used chemotherapeutic agents. They induce DNA crosslinking and activate DDR pathways (Choi and Kim, 2006). However, resistance severely limits their clinical efficacy. Key resistance mechanisms include enhanced DNA repair, increased DDR protein activity, and apoptosis evasion (Choi and Kim, 2006). Recent studies show that NAT10 regulates PARP1 acetylation and DNA repair complex assembly, contributing to platinum resistance. Remodelin reverses this effect. In breast cancer MCF-7 cells, NAT10 overexpression does not significantly affect invasiveness alone (Qi et al., 2022). However, in the presence of platinum drugs, it increases invasion and resistance. NAT10 knockdown enhances drug sensitivity and reduces invasion. Mechanistically, NAT10 interacts with PARP1 and promotes its acetylation, increasing protein stability (Qi et al., 2022). Acetylated PARP1 recruits XRCC1 and LIG3, enhancing base excision repair and single-strand break repair. This improves DNA repair capacity. Under platinum treatment, NAT10 overexpression reduces γH2AX levels, indicating reduced DNA damage (Qi et al., 2022). NAT10 knockdown increases γH2AX, indicating DNA damage accumulation. Remodelin disrupts NAT10–PARP1 interaction, impairs repair complex assembly, and restores DNA damage, thereby reversing platinum resistance.

### 3.6 Remodelin to overcome sorafenib resistance

Sorafenib is a multi-kinase inhibitor used for HCC and other advanced cancers. It also induces ferroptosis. However, resistance limits its therapeutic efficacy (Tang et al., 2020). Resistance is associated with ferroptosis suppression, enhanced antioxidant capacity, and stress adaptation (Tang et al., 2020). Recent studies show that NAT10 regulates ferroptosis-related genes through ac4C modification and contributes to sorafenib resistance. Remodelin restores ferroptosis sensitivity. In nasopharyngeal carcinoma (NPC), NAT10 upregulates SLC7A11 through ac4C modification. SLC7A11 is a key inhibitor of ferroptosis (Xue et al., 2024). It maintains glutathione levels and reduces lipid peroxidation, thereby blocking ferroptosis. NAT10 increases SLC7A11 mRNA stability, suppresses lipid peroxidation, and promotes resistance. NAT10 inhibition restores ferroptosis and increases sorafenib sensitivity (Xue et al., 2024). In resistant models, NAT10 loss suppresses tumor growth and restores drug response. Oxidative stress, such as nanoplastic exposure, also increases NAT10 expression and enhances ac4C modification in tRNA, improving antioxidant capacity (Fu et al., 2025).

### 3.7 Remodelin enhances immune checkpoint blockade

Immune checkpoint blockade (ICB), including anti-PD-1 and anti-PD-L1 antibodies, is an important cancer therapy (Jin et al., 2024). It restores T cell activity by blocking immune suppression. However, its efficacy is often limited by an immunosuppressive tumor microenvironment (Jin et al., 2024). Recent studies show that NAT10 regulates the angiogenesis–immune suppression axis through ac4C RNA modification, contributing to ICB resistance. Remodelin has been reported to improve immunotherapy response in preclinical models, potentially through NAT10-associated remodeling of the tumor microenvironment. In gastric cancer, NAT10 stabilizes lncRNA XIST via ac4C modification (Lei et al., 2025). XIST promotes YAP1 nuclear translocation through hnRNPK recruitment, activating TEAD4-dependent transcription and increasing VEGFA expression (Lei et al., 2025). This leads to abnormal angiogenesis and immune suppression. NAT10 inhibition reduces VEGFA secretion, normalizes tumor vasculature, and improves immune cell infiltration. It also increases CXCL9/10/11 expression, enhances CD8^+^ T cell infiltration, and reduces Treg cells. Combination therapy with Remodelin and YAP1 inhibitors or anti-PD-1 antibodies shows strong synergistic effects and significantly suppresses tumor growth.

### 3.8 NAT10-mediated ac4C regulation in the tumor microenvironment

Another important consideration is that NAT10-targeted strategies may influence therapeutic tolerance not only through direct effects on malignant cells but also by reshaping dynamic components of the tumor microenvironment (Chen et al., 2023). Drug resistance is increasingly recognized as a multicellular process involving reciprocal interactions among cancer cells, stromal cells, immune cells, endothelial cells, and extracellular matrix components (Chen et al., 2023). In this context, NAT10-mediated ac4C modification may regulate stress-adaptive transcripts and signaling programs that affect cytokine production, immune evasion, stromal activation, angiogenesis, and metabolic communication within the tumor niche (Li G. et al., 2024). For example, altered NAT10 activity in tumor cells may influence the secretion of inflammatory mediators, chemokines, or metabolic factors that recruit immunosuppressive myeloid cells, polarize macrophages, impair cytotoxic T-cell function, or activate cancer-associated fibroblasts (Li G. et al., 2024). Conversely, stromal and immune-derived signals may further enhance NAT10-dependent RNA regulatory programs in cancer cells, thereby reinforcing a therapy-tolerant state. Therefore, the therapeutic consequences of NAT10 inhibition should be evaluated within the broader ecological context of the tumor microenvironment rather than solely in cancer cell-intrinsic models. Future studies integrating single-cell profiling, spatial transcriptomics, immune-competent tumor models, and patient-derived samples will be essential to determine how targeting NAT10 and ac4C-regulated RNA networks remodels stromal–immune interactions and whether such remodeling can improve responses to chemotherapy, targeted therapy, radiotherapy, or immunotherapy.

### 3.9 Epitranscriptomic crosstalk between NAT10/ac4C and other RNA modifications

Although this review focuses on NAT10-mediated ac4C, cancer drug resistance is shaped by a broader epitranscriptomic network involving multiple RNA modifications, including m6A, m5C, pseudouridine, and A-to-I RNA editing. These modifications regulate overlapping processes such as RNA stability, splicing, translation, metabolism, DNA damage repair, EMT, and immune evasion. Therefore, NAT10 should be considered not only as an ac4C writer but also as a potential regulatory component within a wider RNA modification landscape. Recent studies have revealed functional crosstalk between NAT10/ac4C and m6A-related pathways. In gastric cancer, NAT10 forms phase-separated condensates and acetylates SRSF2, thereby increasing SRSF2 stability. Acetylated SRSF2 promotes exon 4 skipping of the m6A reader YTHDF1, generating a short YTHDF1 transcript that enhances tumor proliferation, migration, invasion, organoid growth, and disease progression. Clinically, NAT10 expression, SRSF2 expression, and YTHDF1 exon 4 skipping are associated with aggressive gastric cancer phenotypes (Liu et al., 2024c). In osteosarcoma, NAT10-mediated ac4C promotes the stability and translation of the m6A reader YTHDC1. YTHDC1 then recognizes m6A-modified glycolytic transcripts, including LDHA and PFKM, linking the NAT10/ac4C–YTHDC1/m6A axis to metabolic reprogramming and tumor progression (Mei et al., 2024). In adenoid cystic carcinoma, MYB transcriptionally activates NAT10, while NAT10 stabilizes IGF2BP3 mRNA through ac4C modification. IGF2BP3, in turn, stabilizes NAT10 mRNA in an m6A-dependent manner, forming a MYB–NAT10–IGF2BP3 positive feedback loop that promotes MAPK signaling, tumor growth, migration, and invasion (Gao et al., 2026). In contrast, direct mechanistic links between NAT10/ac4C and m5C, pseudouridine, or A-to-I editing remain poorly characterized. However, these modifications also regulate RNA metabolism and cancer-related phenotypes, including stress adaptation, immune recognition, and therapeutic response. Future studies integrating ac4C profiling with m6A, m5C, pseudouridine, and RNA-editing analyses in patient-derived and drug-resistant models will be important to clarify how these RNA modification systems cooperate or compete during treatment resistance.

To provide a clearer overview of the currently available evidence, we summarized representative studies linking NAT10 to cancer drug resistance across different tumor types in Table 1. This table compares the major NAT10-regulated targets or pathways, the corresponding molecular mechanisms, the relevant therapeutic contexts, and the level of supporting evidence. Such a comparison highlights that NAT10 does not act through a single universal mechanism, but instead regulates distinct tumor-dependent programs, including DNA damage repair, metabolic adaptation, EMT, ferroptosis, cancer stemness, and immune evasion. Importantly, most evidence remains preclinical, with only limited clinical association data, underscoring the need for further validation in patient-derived models and prospective clinical studies.

**TABLE 1:** Summary of NAT10-associated mechanisms, therapeutic relevance, and evidence levels in cancer drug resistance.

Tumor type	NAT10 target/regulated axis	Molecular mechanism	Relevant therapy	Level of evidence	Ref.
Ovarian cancer	PGAM1	NAT10-mediated ac4C stabilizes PGAM1 mRNA, enhances glycolysis, and supports cancer stemness	Potential chemotherapy sensitization	*In vitro*	Zhang and Shen (2025)
Colon cancer	NANOGP8	NAT10 stabilizes NANOGP8 mRNA through ac4C modification, maintaining stemness and chemoresistance	Chemotherapy	*In vitro*	Gao et al. (2024)
Bladder cancer	AHNAK/DDR pathway	Cisplatin-induced NF-κB activation upregulates NAT10; NAT10 stabilizes AHNAK mRNA and enhances DNA damage repair	Cisplatin; Remodelin plus cisplatin	*In vitro*, organoid, *in vivo*, clinical association	Xie et al. (2023a)
UV-induced damage/skin tumor model	DDB2	NAT10 regulates DDB2 mRNA stability and nucleotide excision repair in a context-dependent manner	DNA-damaging stress/UV-related tumorigenesis	*In vitro*, *in vivo*	Yang et al. (2023)
Hepatocellular carcinoma and HR-dependent tumors	DNA:RNA hybrids/HR repair	NAT10 is recruited to DNA double-strand break sites in a PARP1-dependent manner and stabilizes DNA:RNA hybrids to promote homologous recombination	PARP inhibitors; Remodelin plus PARP inhibitors	*In vitro*, *in vivo*	Xu et al. (2025)
Triple-negative breast cancer	RAD51	NAT10 stabilizes RAD51 mRNA through ac4C modification, restoring homologous recombination and reducing PARP inhibitor sensitivity	Olaparib/PARP inhibitors	*In vitro*, *in vivo*	Li et al. (2025b)
Colorectal cancer	MYC–PPAN axis	NAT10 enhances MYC and PPAN translation through ac4C modification, strengthening DNA damage repair capacity	DNA-damaging therapies	*In vitro*, *in vivo*, clinical association	Wang et al. (2026)
Pancreatic ductal adenocarcinoma	LAMB3/FAK–ERK/PD-L1	NAT10 stabilizes LAMB3 mRNA, activates FAK/ERK signaling, increases PD-L1 expression, and promotes immune evasion	Immune checkpoint blockade	*In vitro*, *in vivo*, clinical association	Chen et al. (2025)
Cervical cancer	FOXP1/GLUT4/KHK	NAT10 enhances FOXP1 translation and glycolytic metabolism, increasing lactate-mediated immunosuppression	Potential immunotherapy and metabolic therapy combinations	*In vitro*, *in vivo*, clinical association	Chen et al. (2023)
Prostate cancer	CD8^+^ T-cell activity; CCL25/CCR9 axis	NAT10 promotes an immunosuppressive microenvironment and reduces antitumor T-cell activity	Immune checkpoint blockade	*In vitro*, *in vivo*, clinical association	Liu et al. (2024a)
Multiple tumor models	MYC/CDK2/DNMT1/type I IFN pathway	NAT10 suppresses dsRNA accumulation and type I interferon signaling, contributing to immune-cold tumor states	PD-1 blockade; NAT10 inhibition plus immunotherapy	*In vitro*, *in vivo*	Liu et al. (2025)
Acute myeloid leukemia	SLC1A4; HOXA9/MENIN; serine metabolism	NAT10-mediated ac4C reprograms serine uptake and synthesis, supporting leukemogenesis and stemness	Metabolic vulnerability; NAT10 inhibition	*In vitro*, *in vivo*, patient data	Zhang et al. (2024)
Osteosarcoma	ATF4/ASNS/asparagine biosynthesis	NAT10 stabilizes ATF4 mRNA and activates ASNS-mediated asparagine synthesis, supporting survival under metabolic stress	NAT10 inhibition; metabolic therapy	*In vitro*, *in vivo*, clinical association	Zou et al. (2024)
Prostate cancer	HMGA1 and KRT8	NAT10 acetylates and stabilizes HMGA1 and KRT8 mRNAs, promoting cell-cycle progression, EMT, growth, and metastasis	Potential anti-metastatic NAT10-targeted therapy	*In vitro*, *in vivo*, clinical association	Li et al. (2024)
Gastric cancer	AKT signaling/EMT markers	NAT10 inhibition reduces AKT signaling, suppresses proliferation and invasion, increases E-cadherin, and decreases N-cadherin and Vimentin	Remodelin; potential chemotherapy combinations	*In vitro*	Li et al. (2024)
Breast cancer	Twist-dependent EMT	NAT10 inhibition by Remodelin reverses doxorubicin-induced EMT and restores drug sensitivity	Doxorubicin; Remodelin plus doxorubicin	*In vitro*	Wu et al. (2018)
Hepatocellular carcinoma	EMT-related pathways	NAT10 promotes EMT and doxorubicin resistance; inhibition enhances doxorubicin sensitivity	Doxorubicin; Remodelin plus doxorubicin	*In vitro*, *in vivo*	Zhang et al. (2019)
Non-small cell lung cancer	FATP4 and CPT1A/lipid metabolism	NAT10 stabilizes lipid metabolism-related transcripts through ac4C, enhancing fatty acid uptake and β-oxidation under EGFR inhibition	EGFR-TKIs; Remodelin plus EGFR-TKIs	*In vitro*, *in vivo*, clinical association	Fang et al. (2025)
Breast cancer	PARP1 acetylation/XRCC1–LIG3 repair complex	NAT10 acetylates and stabilizes PARP1, enhancing DNA repair complex assembly and platinum resistance	Platinum drugs; Remodelin plus platinum therapy	*In vitro*	Qi et al. (2022)
Nasopharyngeal carcinoma	SLC7A11/ferroptosis pathway	NAT10 stabilizes SLC7A11 mRNA through ac4C modification, suppressing ferroptosis and promoting sorafenib resistance	Sorafenib; Remodelin plus sorafenib	*In vitro*, *in vivo*	Xue et al. (2024)
Oxidative stress-associated resistant models	ROS–NAT10–ac4C axis	Oxidative stress increases NAT10/ac4C activity and enhances antioxidant capacity, contributing to chemoresistance	Sorafenib; NAT10 inhibition	*In vitro*	Fu et al. (2025)
Gastric cancer	XIST/YAP1/VEGFA axis	NAT10 stabilizes XIST *via* ac4C modification, promotes YAP1 nuclear translocation and VEGFA expression, causing vascular abnormalization and immune suppression	Anti-PD-1 therapy; Remodelin plus ICB or YAP1 inhibition	*In vitro*, *in vivo*	Lei et al. (2025)
Head and neck squamous cell carcinoma	NAT10-associated risk pathways	High NAT10 expression is associated with MYC, E2F, G2/M checkpoint, mTORC1, DNA repair, and oxidative phosphorylation pathways; Remodelin inhibits tumor growth in PDX models	NAT10 inhibition; potential chemotherapy or targeted combinations	*In vitro*, PDX, clinical association	Tao et al. (2021)
Gastric cancer	NAT10–SRSF2–YTHDF1 splicing axis	NAT10 phase separation promotes SRSF2 acetylation and YTHDF1 exon 4 skipping, linking ac4C/NAT10 activity with m6A-reader regulation	Potential NAT10/SRSF2/YTHDF1-targeted therapy	*In vitro*, organoid, *in vivo*, clinical association	Liu et al. (2024c)
Osteosarcoma	NAT10/ac4C–YTHDC1/m6A–LDHA/PFKM axis	NAT10 regulates YTHDC1 stability and translation, linking ac4C to m6A-dependent glycolytic regulation	NAT10 inhibition; metabolic therapy	*In vitro*, *in vivo*, clinical association	Mei et al. (2024)
Adenoid cystic carcinoma	MYB–NAT10–IGF2BP3 circuit	MYB upregulates NAT10; NAT10 stabilizes IGF2BP3 mRNA via ac4C, while IGF2BP3 stabilizes NAT10 mRNA in an m6A-dependent manner	Remodelin; NAT10/IGF2BP3-targeted therapy	*In vitro*, *in vivo*, clinical bioinformatics	Gao et al. (2026)

### 3.10 Strength and limitations of current evidence

Although accumulating studies suggest that NAT10 and NAT10-mediated ac4C modification participate in cancer drug resistance, the current evidence should be evaluated with caution. Most available findings are derived from cancer cell lines, xenograft models, genetically engineered mouse models, or limited patient-derived experimental systems, whereas direct clinical validation remains insufficient. These preclinical models are valuable for defining molecular mechanisms, such as regulation of RNA stability, translation, DNA damage repair, metabolic adaptation, EMT, and immune-related pathways. However, they do not fully reproduce the genetic heterogeneity, immune complexity, stromal interactions, pharmacokinetic variability, prior treatment exposure, and evolutionary resistance trajectories observed in patients. In addition, many studies rely on NAT10 knockdown, overexpression, or Remodelin treatment, but these approaches have important methodological limitations. Genetic perturbation may produce non-physiological effects, while pharmacological inhibition may be influenced by incomplete selectivity, off-target activity, dose-dependent toxicity, and differences between short-term experimental exposure and clinically achievable drug concentrations. Furthermore, several reported mechanisms have been established in specific tumor types or experimental contexts, and their generalizability across cancers remains uncertain. Therefore, NAT10 should not be interpreted as a universal driver of therapy resistance, but rather as an important regulatory factor whose contribution may vary by tumor type, molecular background, treatment modality, and microenvironmental context. Future studies should incorporate larger clinical cohorts, patient-derived organoids, immune-competent models, single-cell and spatial profiling, pharmacokinetic/pharmacodynamic analyses, and biomarker-guided validation to better define the clinical relevance, predictive value, and therapeutic feasibility of NAT10-targeted strategies.

## 4 Challenges and future perspectives of Remodelin as an anticancer agent

Remodelin, as a small-molecule inhibitor of NAT10, has shown strong potential in multiple tumor models for reversing drug resistance and enhancing sensitivity to chemotherapy and immunotherapy. It exhibits broad synergistic effects, particularly in combination with cisplatin, PARP inhibitors, EGFR-TKIs, and immune checkpoint blockade therapies. However, its translation from basic research to clinical application still faces several challenges.

### 4.1 Current limitations

As an inhibitor of NAT10, Remodelin may exhibit non-specific effects in different cellular contexts. Its potential interference with other acetyltransferases or RNA modification-related proteins has not been fully clarified. This limits mechanistic precision and complicates evaluation of clinical safety. NAT10 is involved in fundamental RNA modification and maintenance of cellular homeostasis. Its broad expression in normal tissues suggests that long-term or systemic inhibition may cause cytotoxic effects (Xie L. et al., 2023). This concern is particularly relevant in highly proliferative tissues, where normal physiological functions may be affected. At present, Remodelin research remains largely at the cellular and animal levels. Systematic clinical trial data are lacking. Its pharmacokinetics, tissue distribution, and long-term safety profile remain unclear (Xie L. et al., 2023). NAT10 modifies multiple RNA substrates, including mRNA, tRNA, and lncRNA, through ac4C modification. However, the functional contribution of different substrates varies across tumor types. This results in context-dependent biological outcomes after inhibition. NAT10 may interact with other RNA modification systems such as m6A. Compensatory or crosstalk mechanisms may be activated after NAT10 inhibition, potentially leading to adaptive resistance and reduced long-term efficacy. Existing studies suggest that NAT10 may be regulated by NF-κB, p300, and metabolic stress signals (Gu et al., 2024). Whether inhibition of NAT10 triggers feedback activation of alternative survival pathways, such as stress adaptation or compensatory repair mechanisms, remains to be further investigated.

### 4.2 Potential consequences of long-term NAT10 inhibition by Remodelin

Although Remodelin has been widely used as a pharmacological tool to inhibit NAT10 activity and explore the therapeutic relevance of NAT10-mediated ac4C regulation, the potential consequences of long-term NAT10 inhibition remain insufficiently defined. This issue is particularly important because cancer progression and recurrence are dynamic processes shaped by clonal evolution, adaptive stress responses, residual disease persistence, and microenvironmental remodeling. Sustained NAT10 inhibition may suppress tumor growth and reduce therapy-tolerant phenotypes by impairing ac4C-dependent RNA stability, translation, and stress-adaptive gene expression (Wang Y. et al., 2025). However, prolonged inhibition could also impose selective pressure on tumor cells, allowing the emergence of resistant subclones or compensatory pathways that bypass NAT10-dependent regulation. In addition, because NAT10 participates in multiple biological processes beyond mRNA acetylation, including ribosomal RNA/tRNA modification, chromatin organization, DNA damage responses, and cellular homeostasis, chronic NAT10 blockade may have complex effects on both malignant and normal tissues (Zhong et al., 2026). These considerations suggest that Remodelin-based NAT10 targeting should not be viewed solely as an acute anti-tumor intervention, but also as a strategy whose long-term impact on tumor dormancy, minimal residual disease, metastatic outgrowth, and disease relapse requires careful evaluation. Future studies should therefore assess the durability of treatment response, mechanisms of acquired resistance, recurrence patterns after drug withdrawal, and potential toxicities associated with prolonged NAT10 inhibition. Such investigations will be essential for determining whether Remodelin or more selective NAT10 inhibitors can be safely and effectively integrated into long-term cancer treatment strategies.

### 4.3 Tumor heterogeneity and differential responses to NAT10 inhibition

Tumor heterogeneity should be carefully considered when evaluating the therapeutic potential of NAT10 inhibition. NAT10 expression and NAT10-mediated ac4C regulatory activity may vary substantially across tumor types, molecular subtypes, disease stages, metastatic sites, and even among different cellular populations within the same tumor (Jiang et al., 2024). Such heterogeneity may directly influence the dependence of cancer cells on NAT10-driven RNA regulatory programs and thereby shape their sensitivity or resistance to NAT10 inhibition (Jiang et al., 2024). Importantly, NAT10 does not appear to regulate a single universal resistance pathway in all cancers. Instead, different tumors may depend on distinct NAT10-associated programs according to their lineage-specific transcriptional state, oncogenic drivers, metabolic requirements, DNA repair capacity, immune microenvironment, and dominant mechanisms of therapeutic pressure.

For example, tumors exposed to DNA-damaging therapies may be more dependent on NAT10-mediated regulation of DNA damage repair and stress-response transcripts, whereas metabolically flexible tumors may rely more heavily on NAT10-regulated amino acid or glycolytic pathways. Highly invasive or metastatic tumors may be more sensitive to NAT10 effects on EMT-related transcripts and signaling pathways, while immune-inflamed or immune-evasive tumors may display greater dependence on NAT10-associated immune regulatory programs (Chen et al., 2024). In addition, the functional consequences of NAT10 inhibition may differ depending on whether NAT10 primarily regulates RNA stability, translation efficiency, protein acetylation, phase separation, or crosstalk with other RNA modification systems such as m6A (Mei et al., 2024). These context-dependent mechanisms may explain why NAT10 inhibition produces stronger effects in some cancer models than in others.

Tumors with high NAT10 expression or strong activation of ac4C-dependent RNA stability and translation programs may be more vulnerable to NAT10-targeted strategies, whereas tumors with low NAT10 expression, redundant RNA-modification pathways, or compensatory survival mechanisms may show limited response (Long et al., 2023). In addition, intratumoral heterogeneity may allow NAT10-independent subclones to survive treatment and contribute to minimal residual disease, adaptive resistance, or recurrence (Long et al., 2023). Therefore, conclusions regarding NAT10 inhibition should not be generalized across all cancers without considering tumor-specific and patient-specific contexts.

### 4.4 Future perspectives: pharmacological limitations, translational challenges, and combination strategies

Although Remodelin has been widely used as a pharmacological tool to investigate NAT10-dependent biological processes, its current therapeutic relevance should be interpreted with caution. Most available evidence supporting the anti-tumor or drug-sensitizing effects of Remodelin is derived from cell-based assays and animal models, and there is currently no clinical evidence demonstrating its efficacy or safety in patients with cancer. Therefore, the findings obtained with Remodelin should be regarded primarily as preclinical observations rather than direct evidence of clinical applicability. Several obstacles must be addressed before NAT10 inhibition can be translated into therapeutic strategies, including the limited understanding of Remodelin pharmacokinetics and pharmacodynamics, potential off-target effects, uncertain selectivity for NAT10, possible toxicity in normal proliferating tissues, and the lack of validated biomarkers to identify patients who may benefit from NAT10-targeted treatment. In addition, optimal dosing schedules, tumor-type specificity, and rational combination regimens with chemotherapy, targeted therapy, radiotherapy, or immunotherapy remain to be systematically evaluated. Future studies using clinically relevant models, patient-derived samples, and eventually well-designed clinical trials will be essential to determine whether Remodelin or more selective NAT10 inhibitors can be developed into effective therapeutic agents.

Rational combination strategies may represent a promising direction for future NAT10-targeted therapy. Because cancer drug resistance is rarely driven by a single pathway, combining Remodelin or more selective NAT10 inhibitors with established anticancer treatments may help suppress compensatory adaptation and improve treatment durability (Larrieu et al., 2014). Preclinical studies have suggested potential synergy between Remodelin and several therapeutic modalities, including cisplatin, PARP inhibitors, EGFR-TKIs, doxorubicin, radiotherapy, and immune checkpoint blockade. Mechanistically, these combinations may involve impaired DNA damage response, reduced stress tolerance, altered RNA stability and translation of resistance-associated genes, reversal of EMT-related plasticity, disruption of metabolic adaptation, and remodeling of the tumor immune microenvironment. In the context of immunotherapy, NAT10 inhibition may influence tumor-intrinsic immune-evasion programs, antigen-presentation machinery, inflammatory signaling, CD8^+^ T-cell infiltration, and immunosuppressive components such as regulatory T cells (Chen et al., 2023). Future studies should systematically evaluate NAT10 inhibition in combination with chemotherapy, radiotherapy, targeted therapy, and immune checkpoint inhibitors, with careful attention to tumor-type specificity, dosing schedules, treatment sequencing, synergistic efficacy, toxicity, and biomarkers predicting response.

Despite these opportunities, several barriers must be overcome before NAT10-targeted therapy can be advanced toward clinical application. Remodelin has been widely used as a pharmacological tool to investigate NAT10-related biology, but its selectivity remains incompletely defined, and some reported effects may involve off-target or NAT10-independent mechanisms (Wang et al., 2022; Mo et al., 2025). Other NAT10-targeting compounds, such as fludarabine, have also been reported to interfere with NAT10-related vulnerabilities, supporting the druggability of NAT10. However, the development of next-generation inhibitors with improved potency, specificity, pharmacokinetic properties, tumor selectivity, and safety profiles will be essential. In parallel, NAT10 expression, ac4C modification patterns, or NAT10-dependent transcriptional and translational signatures may serve as potential biomarkers to identify patients most likely to benefit from NAT10-targeted interventions.

### 4.5 Pharmacological limitations of Remodelin

Although Remodelin is widely used as a pharmacological inhibitor of NAT10, it should not be regarded as a fully validated NAT10-specific therapeutic agent. Current evidence regarding its selectivity remains limited, and some biological effects observed after Remodelin treatment may reflect off-target or NAT10-independent mechanisms rather than exclusive inhibition of NAT10. This issue is particularly important because NAT10 participates in diverse cellular processes, while Remodelin may influence broad stress-response pathways, RNA metabolism, chromatin-associated functions, DNA damage responses, and cell-cycle regulation (Yu et al., 2025). Therefore, conclusions based solely on Remodelin treatment should be interpreted cautiously and ideally supported by complementary genetic approaches, such as NAT10 knockdown, knockout, catalytic-mutant rescue, or orthogonal inhibitors.

In addition, the pharmacokinetic and pharmacodynamic properties of Remodelin remain insufficiently characterized in cancer-relevant settings. Key parameters, including bioavailability, tissue distribution, metabolic stability, plasma half-life, tumor penetration, dose-response relationships, and clinically achievable drug concentrations, have not been systematically established (Xie R. et al., 2023). As a result, it remains unclear whether the concentrations used in many cell-line or animal studies can be safely and effectively achieved in patients. Remodelin may also have dose-dependent cytotoxicity or affect normal proliferating cells, especially given the role of NAT10 in RNA acetylation, ribosome biogenesis, chromatin organization, DNA repair, and cellular stress adaptation. These uncertainties limit direct extrapolation from preclinical observations to clinical therapeutic potential.

**FIGURE 2:**
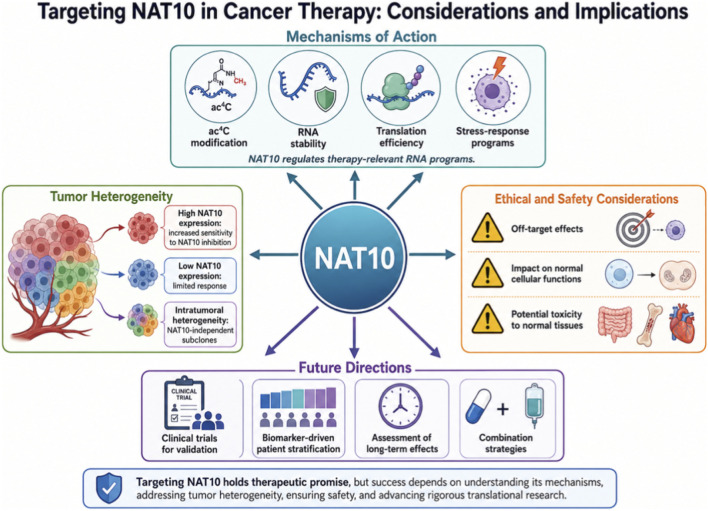
Conceptual framework for targeting NAT10 in cancer therapy. NAT10-mediated ac4C RNA modification represents an emerging post-transcriptional regulatory mechanism involved in tumor progression and therapeutic resistance.

Future studies should therefore define Remodelin’s target spectrum, off-target profile, pharmacokinetics, pharmacodynamics, and toxicity using rigorous chemical biology and preclinical pharmacology approaches. Comparative studies using structurally distinct NAT10 inhibitors, NAT10 catalytic mutants, rescue experiments, and patient-derived models will be important to distinguish NAT10-dependent effects from compound-specific effects. Ultimately, the development of next-generation NAT10 inhibitors with improved potency, selectivity, tumor penetration, metabolic stability, and safety profiles will be necessary before NAT10-targeted therapy can be responsibly advanced toward clinical application. Overall, NAT10-targeted therapy remains a promising but still emerging strategy for overcoming selected forms of cancer drug resistance. Its future development will depend on more selective inhibitors, clinically relevant models, rational combination regimens, robust predictive biomarkers, and well-designed clinical trials. A balanced framework that integrates therapeutic benefit, tumor heterogeneity, normal tissue safety, off-target effects, and clinical validation will be essential to determine whether NAT10-mediated ac4C regulation can be translated into effective and responsible cancer treatment strategies, as summarized in Figure 2.

### 4.6 Biomarker potential of NAT10 and ac4C in clinical translation

NAT10 and NAT10-mediated ac4C modification may have potential value as prognostic and predictive biomarkers, although their clinical application remains preliminary. In head and neck squamous cell carcinoma (HNSCC), NAT10 was identified as a key risk gene and an independent prognostic factor in the TCGA-HNSCC dataset. High NAT10 expression was associated with MYC, E2F, G2/M checkpoint, mTORC1, DNA repair, and oxidative phosphorylation pathways. NAT10 protein expression was also significantly increased in tumor cells compared with normal epithelial cells in FFPE samples, and higher NAT10 levels correlated with poor overall survival in 267 HNSCC patients (Tao et al., 2021). Similarly, in papillary renal cell carcinoma, NAT10 was upregulated in malignant cells, associated with advanced stage and poor prognosis, and incorporated into a prognostic model with strong survival-prediction performance. Higher NAT10-related risk scores were also linked to poorer predicted immunotherapy response (Li L. et al., 2024). These findings suggest that NAT10 may reflect aggressive tumor states characterized by enhanced proliferation, DNA repair, metabolic activity, and tumor microenvironment dysregulation. NAT10 may also have predictive value for treatment response, as NAT10 depletion or Remodelin treatment suppressed tumor growth in HNSCC experimental models (Tao et al., 2021). However, this predictive role remains to be validated in prospective clinical cohorts.

For clinical translation, standardized quantification methods and thresholds are needed. NAT10 can be measured by RNA sequencing, quantitative PCR, or immunohistochemistry in FFPE tissues, whereas ac4C can be assessed by acRIP-seq, LC–MS/MS, dot blot, or site-specific profiling. At present, no universal cutoff defines “NAT10-high” or “ac4C-high” tumors, and most studies rely on cohort-specific median values, survival-based cutoffs, immunohistochemical scores, or computational risk models. Future studies should establish reproducible assays, tumor-type-specific thresholds, and independent validation cohorts to determine whether NAT10/ac4C can guide prognosis, patient stratification, and therapeutic decision-making.

## 5 Conclusion

NAT10 is a major writer enzyme of ac4C RNA epitranscriptomic modification and has been implicated in tumor development and therapy resistance in multiple preclinical studies. Through regulation of mRNA stability and translation efficiency, it links RNA modification with DNA damage repair, metabolic reprogramming, EMT transition, and remodeling of the tumor immune microenvironment. Together, these processes may contribute to tumor adaptive survival and therapeutic escape in a context-dependent manner. Remodelin, as a small-molecule inhibitor commonly used to study NAT10, has been shown in preclinical models to attenuate several resistance-associated phenotypes by interfering with NAT10/ac4C-related regulation. It acts at multiple levels, including weakening DNA repair capacity, blocking metabolic adaptation, reversing EMT phenotypes, and improving the immunosuppressive microenvironment. These observations suggest that NAT10 inhibition may influence multiple resistance-associated pathways, although whether this translates into clinically meaningful benefit remains uncertain. In the future, combination strategies informed by the multi-dimensional regulatory functions of NAT10 may represent a promising but still emerging approach to overcoming cancer drug resistance, particularly in tumor contexts where NAT10 has been mechanistically implicated. These may include combinations with chemotherapy, targeted therapy, immune checkpoint inhibitors, metabolic interventions, and chronotherapy. With improved inhibitor selectivity, biomarker-guided patient stratification, and mechanism-based combination design, NAT10-targeted strategies may provide a useful therapeutic avenue for selected refractory cancers in the future.
